# ﻿A new feather mite species of the genus *Mycterialges* Gaud & Atyeo, 1981 (Acari, Xolalgidae) from the Oriental Stork, *Ciconiaboyciana* (Ciconiiformes, Ciconiidae) in Korea

**DOI:** 10.3897/zookeys.1192.115749

**Published:** 2024-02-21

**Authors:** Jeong Hun Shim, Yeong-Deok Han, Sukyung Kim, Dongsoo Ha, Yongun Shin, Soo Hyung Eo

**Affiliations:** 1 Department of Forest Science, Kongju National University, Yesan, Republic of Korea Kongju National University Yesan Republic of Korea; 2 Research Center for Endangered Species, National Institute of Ecology, Yeongyang, Republic of Korea ﻿Research Center for Endangered Species, National Institute of Ecology ﻿Yeongyang Republic of Korea; 3 Eco-institute for Oriental Stork, Korea National University of Education, Cheongju, Republic of Korea Korea National University of Education Cheongju Republic of Korea; 4 Natural Heritage Division, Cultural Heritage Administration, Deajeon, Republic of Korea ﻿Natural Heritage Division, Cultural Heritage Administration Deajeon Republic of Korea

**Keywords:** Analgoidea, Astigmata, COI, ectosymbionts, Ingrassiinae, systematics, taxonomy

## Abstract

A new feather mite species, *Mycterialgesboycianae***sp. nov.** (Xolalgidae), was identified from the Oriental Stork, *Ciconiaboyciana* Swinhoe, 1873, in Korea. Males of *M.boycianae***sp. nov.** are distinguished from *Mycterialgesmesomorphus* Gaud & Atyeo, 1981, in having a single triangular prodorsal shield, sinuous margins of the opisthosoma located between setae *e*2 and *h*2 on the hysteronotal shield, an oval-shaped epiandrum without posterior extensions, a shorter tibia + tarsus IV than femoragenu IV, and an absent ambulacral disc of leg IV. Females differ in having a prodorsal shield with a posterior margin that is blunt-angular, and a concave posterior margin of the hysteronotal shield with posterior extensions. This study presents the first record of the feather mite genus *Mycterialges* in birds of the genus *Ciconia* (Ciconiidae). Additionally, we determined the phylogenetic relationship among Ingrassiinae using the mitochondrial cytochrome *c* oxidase subunit (COI).

## ﻿Introduction

Feather mites (Astigmata) belong to two superfamilies (Analgoidea and Pterolichoidea), most of which are permanent ectosymbionts. These mites live on specific feathers and microsites on feathers, primarily consuming fungi and bacteria (Doña et al. 2019). They are mainly transmitted vertically from parents to offspring (Doña et al. 2017), and because of these characteristics, they exhibit high host specificity (OConnor 1982; Gaud and Atyeo 1996; Dabert and Mironov 1999; Proctor 2003). Feather mites living on endangered birds could be at risk of extinction due to their high host specificity, as a decrease in the host population could affect them (Waki et al. 2023). For example, *Compressalgesnipponiae* Dubinin, 1950 (Pterolichoidea: Caudiferidae), which is associated with the Crested Ibis *Nipponianippon* (Temminck, 1835), disappeared due to the extirpation of its native host *N.nippon* in Japan. Consequently, this mite was registered as “Extinct” in the Red List of the Ministry of the Environment, Japan in 2020 (Waki and Shimano 2020; Waki et al. 2023).

The genus *Mycterialges* Gaud & Atyeo, 1981 is distinguished from other genera in the family Xolalgidae Dubinin, 1953 (Analgoidea) by the number of setae on the anterior tarsi, ambularcal disc shape, male opisthosomal lobes structure and legs IV, and the female’s epigynum form (Gaud and Atyeo 1981a, 1996). This genus is associated with birds of Ciconiidae; only two species of this genus have been reported (Gaud and Atyeo 1981a, 1996; Gaud 1982). The first discovered type species was *Mycterialgesmesomorphus* Gaud & Atyeo, 1981, which was recorded in the Wood Stork, *Mycteriaamericana* Linnaeus, 1758 in North America. Another species was found in the Saddle-billed Stork, *Ephippiorhynchussenegalensis* (Shaw, 1800) in Africa, but it has not yet been accurately described (Gaud 1982).

The family Ciconiidae consists of 20 species in six genera, which are found on all continents except Antarctica. In Korea, two species of the genus *Ciconia* occur: the Black Stork, *Ciconianigra* (Linnaeus, 1758) and the Oriental Stork, *Ciconiaboyciana* Swinhoe, 1873. Both species are observed as winter visitors and are designated as “Endangered Species Level I” by the Korea Ministry of Environment (BirdLife International 2017, 2018; Lee et al. 2020; Gill et al. 2023; NIBR 2023). Among them, *C.boyciana* has become extirpated from Korea’s breeding population since the 1970s due to overfishing, habitat destruction, and food shortages (Sonobe and Izawa 1987; Chan 1991; Collar et al. 2001; Cheong 2005). In Korea, efforts have been made to restore a breeding population of *C.boyciana* since 1996 by importing Oriental Storks from Germany, Russia and Japan to increase their population. Since 2007, storks have been released into the wild to restore the breeding population (Park et al. 2011; Son et al. 2011; Eco-Institute for Oriental Stork 2023).

Research on the migration routes and nest selection of *C.boyciana* has mainly been conducted in East Asia (Shimazaki et al. 2004; Cheng et al. 2023). However, despite active research on the behavior and ecology of the Oriental Storks, research on ectosymbionts has not been actively conducted. *Pelargolichusorientalis* Waki, Mironov & Shimano, 2023 is the only known feather mite of *C.boyciana*. This mite was previously identified as *Pelargolichusdidactylus* (Trouessart, 1885) (Dubinin 1956). However, Pérez and Atyeo (1992) identified mites in the Oriental Stork as a different species within the genus *Pelargolichus*, which was later described as *P.orientalis* by Waki et al. (2023). Except for this mite, little is known about other mites associated with Oriental Storks. Since 1996, ecological research has been conducted on the feeding behavior, reintroduction suitability, and habitat of Oriental Storks (Cheong 2005; Sung et al. 2008; Kim 2009; Ha 2019). Recently, we started research on ectosymbionts that can affect the host while interacting with storks.

This study reports a new species of feather mite of the genus *Mycterialges* found on captive *C.boyciana* in Korea. We provide information on the external morphology of the newly discovered feather mite species. In addition, we used the mitochondrial DNA (mtDNA) cytochrome *c* oxidase subunit I (COI) sequence information to determine the phylogenetic relationship between the new species and known closely related species.

## ﻿Material and methods

### ﻿Sampling and characterization

Feather mite sampling from three captive *C.boyciana* individuals was conducted in the same cage at Yesan Oriental Stork Park in Korea in October 2022, with permission from the Cultural Heritage Administration of Korea (B0030104016624). To minimize stress during the investigation, the storks were blindfolded and immobilized by the keepers; the procedure was completed within a maximum of five minutes for each individual. A new species of feather mites was identified from the wing and body feathers of the birds. The mites were carefully removed from the feathers using tweezers, and the collected mites were preserved in 95% ethanol solution. The specimens were mounted on microscope slides using a polyvinyl alcohol mounting medium (BioQuip, California, USA) after clearing with 10% lactic acid (Downs 1943; Han et al. 2016). Specimens were examined using a Dhyana 400DC camera (TUCSEN, Fuzhou, Fujian, China), Leica DM 2000 microscope (Leica, Wetzlar, Germany) with a drawing tube.

Descriptions of a new species followed the standard formats proposed for mites of the subfamily Ingrassiinae Gaud and Atyeo 1981 (Mironov and Proctor 2008; Stefan et al. 2013; Hernandes and Pedroso 2017; Han et al. 2021; Mironov and Hribar 2023). General morphological terms followed Gaud and Atyeo (1996) with minor corrections for coxal setation by Norton (1998). The classification and names of the birds followed those described by Gill et al. (2023). All measurements are in micrometres (μm).

### ﻿DNA sequencing and molecular analysis

Genomic DNA was extracted from three specimens of the new feather mite using the DNeasy Blood and Tissue Kit (QIAGEN, Hilden, Germany). The COI barcode fragment was amplified using primer set, bcdF05 (5′-TTTTCTACHAAYCATAAAGATATTGC-3′) and bcdR04 (5′- TATAAACYTCDGGATGNCCAAAAAA-3′) (Dabert et al. 2008). Polymerase chain reaction (PCR) was conducted following the protocol of A-star *Taq* DNA Polymerase (BIOFACT, Daejeon, Korea). The cycle conditions were as follows: 5 min at 95 °C; 40 cycles at 95 °C for 15 sec, 50 °C for 30 sec, and 72 °C for 60 sec; and a final extension at 72 °C for 5 min (Dabert et al. 2008; Han et al. 2021).

Sequence editing including assembly, alignment, and trimming, was performed using the GENEIOUS v.10.2.5 software (Kearse et al. 2012). We obtained a 635–683 bp fragment sequence of the COI gene from three individuals per mite species. Subsequently, the sequences of these three specimens were used for phylogenetic analysis, together with 17 COI regions of the subfamily Ingrassiinae obtained from the National Center for Biotechnology Information (NCBI). In addition, we collected data from the NCBI on one individual of the subfamily Xolalginae Dubinin, 1953 (Gaud and Atyeo 1981a, b, 1996). The samples were aligned using GENEIOUS and the COI fragment was trimmed to 548 bp. A phylogenetic tree was constructed using COI fragments of the 21 feather mites (Table 1) and generated using the maximum-likelihood (ML) algorithm in PhyML v.3.0 (Guindon et al. 2010). To calculate nucleotide substitution, we used the Hasegawa-Kishino-Yano-1985 (HKY85) + gamma distribution and invariant site (G+I) model, which was selected as the best model based on Smart Model Selection (SMS) (Lefort et al. 2017). The reliability of the tree was tested using 1000 bootstrap replicates (Felsenstein 1985).

**Table 1.** Feather mites used for the phylogenetic analysis in this study (COI barcode fragment).

Mite subfamily	Mite species	Host species	Sample source	GenBank accession No.	Reference
Xolalginae (outgroup)	*Xolalgoides* sp.	*Vireohypochryseus*	Mexico	KU203107	Klimov et al. 2017
Ingrassiinae	*Analloptes* sp.	*Xiporhynchusflavigaster*		KU203108	
	*Glaucalges* sp.	*Tytoalba*	Germany	EU271955	Dabert et al. 2008
				EU271956	
	*Glaucalgesattenuatus*	*Asiootus*		EU271957	
				EU271958	
	*Ingrassia* sp.	–	–	EU271954	Unpublishied
		–	–	GQ864347	Dabert et al. 2010
	*Ingrassiachionis*	*Chionisalbus*	Antarctica	MZ489649	Han et al. 2021
				MZ489650	
	*Ingrassiaoceanodromae*	–	–	OL685164	Unpublishied
	*Ingrassiaphilomachi*	*Calidrispugnax*	Kazakhstan	KU203104	Klimov et al. 2017
	*Ingrassiaveligera*	*Tringaglareola*	Korea	MK031706	Han and Min 2019
	*Ingrassiella* sp.	*Catharusfuscater*	Peru	KU203102	Klimov et al. 2017
	*Metingrassiapelecani*	–	–	MG407963	Unpublishied
		–	–	MG408765	
		–	–	MG410544	
	*Mycterialgesboycianae* sp. nov.	*Ciconiaboyciana*	Korea	OR802170	This study
				OR802171	
				OR802172	
	*Vingrassiavelata*	*Anascrecca*	Russia	KU203105	Klimov et al. 2017

## ﻿Results

### ﻿Systematics

**Superfamily Analgoidea Trouessart & Mégnin, 1884**

**Family Xolalgidae Dubinin, 1953**

**Subfamily Ingrassiinae Gaud & Atyeo, 1981**

#### ﻿Genus Mycterialges

Taxon classificationAnimaliaAstigmataXolalgidae

Gaud & Atyeo, 1981

37857476-7BF6-5E7D-B242-17157F31BA0B

##### Type species.

*Mycterialgesmesomorphus* Gaud & Atyeo, 1981, by original designation.

##### Remarks.

To date, the genus *Mycterialges* has included only one described species, *M.mesomorphus*, found on the Wood Stork, *Mycteriaamericana* (Ciconiiformes: Ciconiidae), in Florida, USA (Gaud and Atyeo 1981a). In the review of feather mites associated with ciconiiforms in Africa, Gaud (1982) recognized one more *Mycterialges* species from the Saddle-billed Stork, *Ephippiorhynchussenegalensis* in Uganda. This unnamed species known only from a single male was illustrated but has never been formally described.

#### ﻿ Mycterialges boycianae

Taxon classificationAnimaliaAstigmataXolalgidae

Shim, Han & Eo sp. nov.

9CA4C6B0-503C-5C65-A422-7A9A4A111FDE

https://zoobank.org/FAEA2BCB-5781-4E20-B15B-5B46F77DCC34

##### Type material.

**Male *holotype*** (Prof. Eo lab, Kongju National University no. ESH_Em00001), and two male and three female ***paratypes*** (Prof. Eo lab no. ESH_Em00002-ESH_Em00006) from wing coverts and plumages of *Ciconiaboyciana* (Ciconiiformes: Ciconiidae), Korea, Chungcheongnam-do, Yesan-gun, Yesan Oriental Stork Park, 36°32'32"N, 126°48'08"E, 17 October 2022, coll. by Shim JH and Han Y.-D.

##### Description.

**Figure 1.**
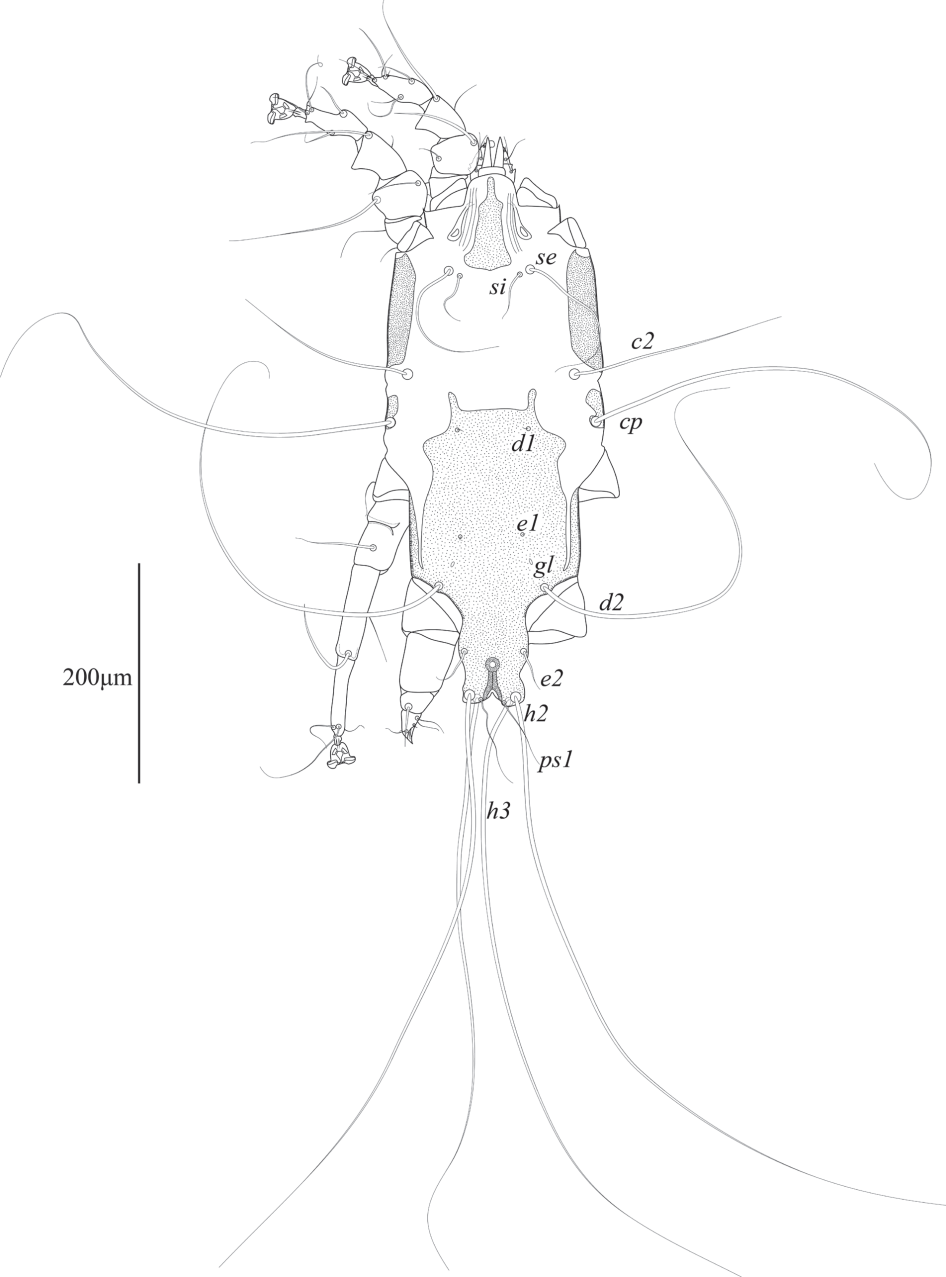
*Mycterialgesboycianae* sp. nov., male dorsal view.

**Figure 2.**
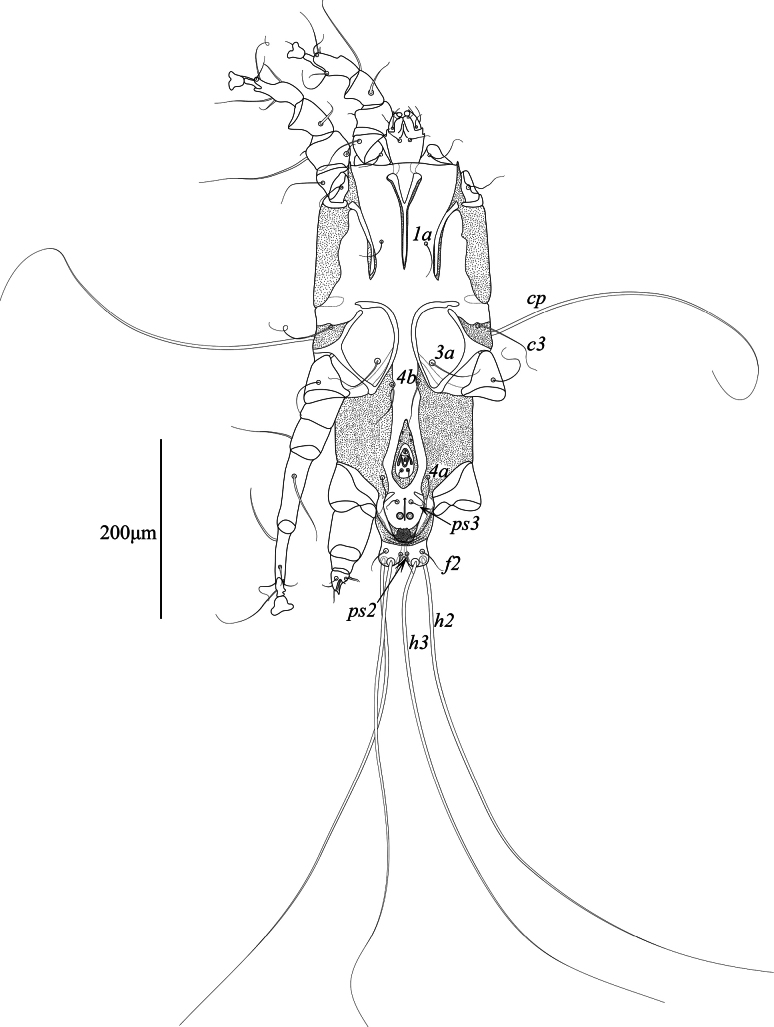
*Mycterialgesboycianae* sp. nov., male ventral view.

**Male** (holotype, range for 2 paratypes in parentheses) (Figs 1, 2, 5A–E). Length of idiosoma from anterior end to bases of setae *h3* 460 (465–470), greatest width 200 (210–220), length of hysterosoma 290 (285–300). Lateral margins of subcapitulum lateral margins with small tooth-like extensions. Prodorsal shield: narrow triangular plate occupying median part of prodorsum, posterior margin slightly convex and almost extending to level of scapular setae *se*, 81 (78–79) in length along midline, 45 (43–45) in width in posterior part (Fig. 1). Setae *se* separated by 75 (74–76), setae *si* situated slightly posterior to level of setae *se*. Scapular shields wide, with inner margin almost straight. Setae *c2* represented by macrosetae, 370 (280–290) long, situated on soft tegument. Humeral shields well developed, fused ventrally with epimerites III. Humeral setae *cp* 390 (290–310) long, situated on posterior margins; setae *c3* filiform, 93 (62–71) long, situated ventrally on anterior margin of humeral shields. Hyteronotal shield: anterior margin straight, anterior angles represented by narrow finger-like extensions anterior to setae *d1*, lateral margins with rounded convexities posterior to level of setae *cp*, length of shield from tips of anterior extensions to bases of setae *h3* 280 (270–275) (Fig. 1). Setae *d2* represented by macrosetae, 350 (270–345) in length. Opisthosoma nearly as wide as one-third of anterior part of hysterosoma, lateral margins of opisthosoma between levels of setae *e2* and *h2* shallowly concave, width at level of setae *h2* 53 (45–52). Supranal concavity circular, separated from the terminal cleft. Terminal cleft small angular, 12 (12–13) long. Opisthosomal lobes fused to each other in basal part and separated by median sclerotized septum, free parts of lobes represented by short and rounded convexities on posterior margin of opisthosoma. Setae *ps1* situated at level of setae *h2*. Distance between dorsal setae: *c2:e1* 140 (130–140), *e1:d2* 48 (43–49), *d2:h3* 100 (95–100), *h3:h3* 27 (26–29), *ps1:ps1* 14 (15).

Epimerites I fused into a Y with stem about 2/3 the length of epimerites. Coxal fields I–II open; coxal fields III almost closed, with small gap in anterior end. Coxal fields IV completely sclerotized, with posterolateral angles fused with lateral margins of hysteronotal shields. Setae *4a* and *4b* situated on inner margins of sclerotized coxal fields IV. Coxal setae *4b* posterior to level of setae *3a*. Genital apparatus 21 (20–21) long and 19 (18–20) wide, with base situated at level of anterior margin of trochanters IV. Paragenital apodemes fused into large teardrop-shaped sclerite 72 (72–78) long and 30 (27–32) wide, encircling genital apparatus and setae *g*. Genital papillae situated on anterior part of fused paragenital apodemes. Opisthoventral shields fused to each other at midline forming entire shield flanking anal field posterolaterally. Diameter of adanal suckers 7 (7–8). Distance between ventral setae: *4b:4b* 30 (38–43), *4b:3a* 21 (17–18), *4b:g* 95 (81–86), *g:ps3* 37 (40–41), *ps3:h3* 70 (62–72).

Tarsi I, II without apico-dorsal extension. Ventral setae *la*, *s* of tarsi I and setae *la*, *wa* and *s* of tarsi II absent. Tibia I, II with spine-like ventral processes. Leg IV short and thickened, with tibia and tarsus extending beyond lobar apices. Length of tibia IV along external margin 19 (18–22). Tarsus IV conical, 25 (24–26) long, ambulacral disc absent, ambulacral stalk acute apically, setae *d* and *e* of tarsus IV filiform and spine, respectively. Length of solenidia: *σ* I 90 (64–77), *σ*II 47 (43–46), *σ*III 68 (63–64), *φ*I 100 (93–94), *φ*II 105 (77–90), *φ*III 87 (83–88), *φ*IV 33 (32–32).

**Figure 3.**
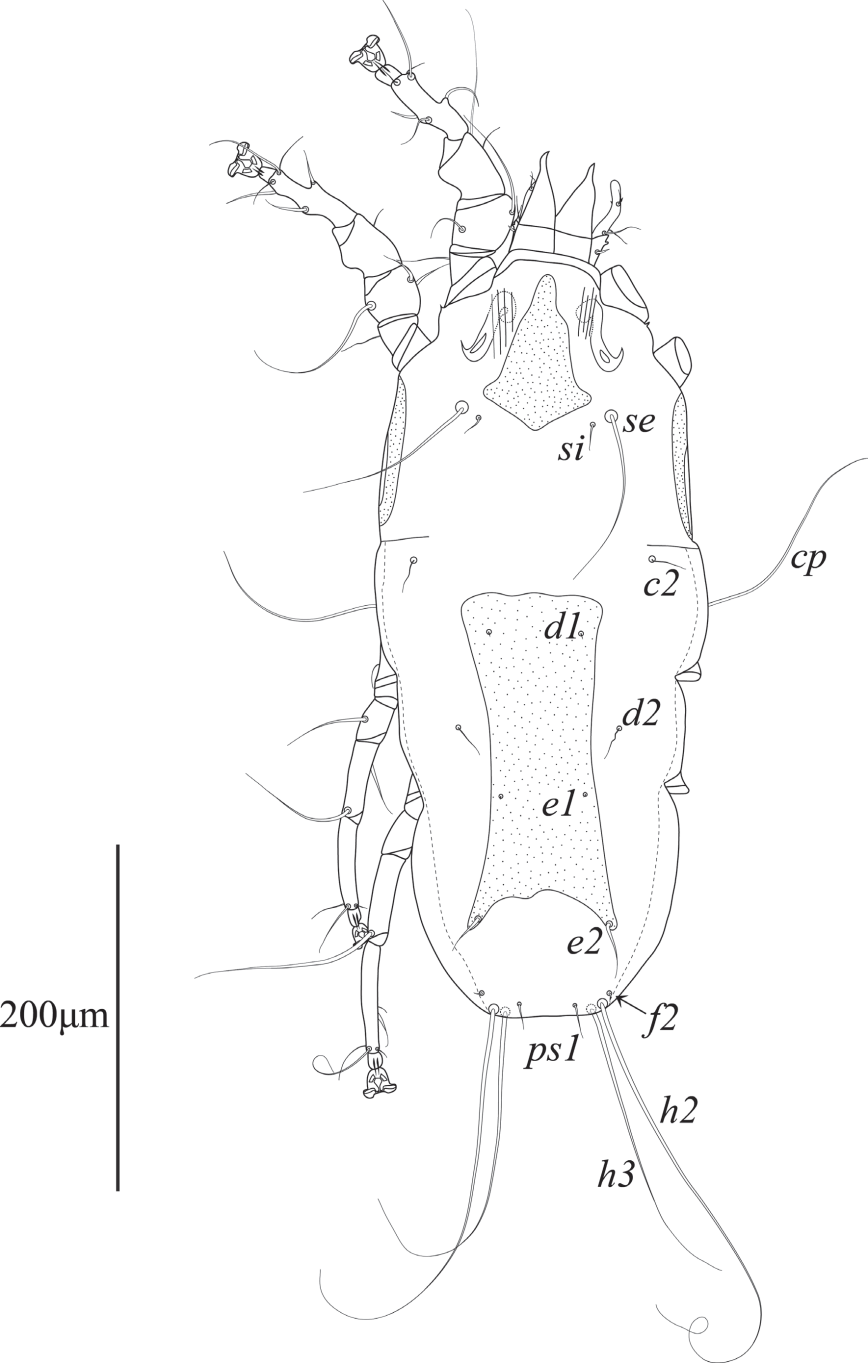
*Mycterialgesboycianae* sp. nov., female dorsal view.

**Figure 4.**
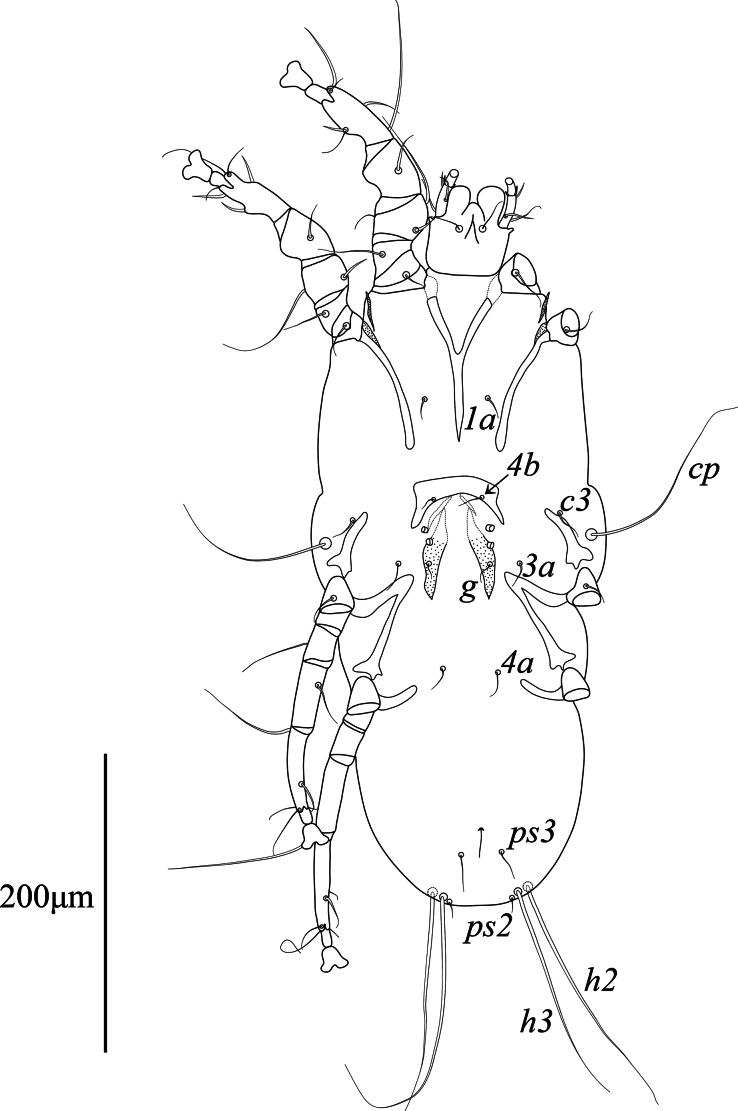
*Mycterialgesboycianae* sp. nov., female ventral view.

**Figure 5.**
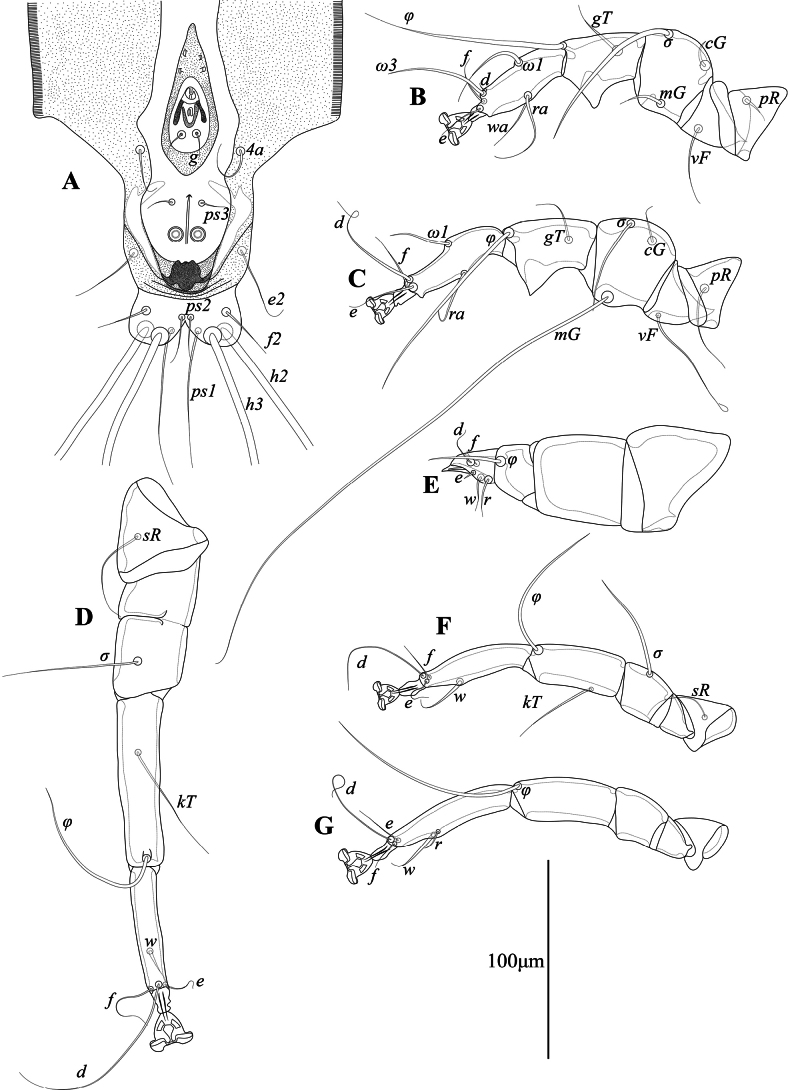
*Mycterialgesboycianae* sp. nov., details, ventral view **A** opisthoma of male, dorsal view **B** leg Ι of male **C** leg II of male **D** leg III of male **E** leg IV of male **F** tibia and tarsus III of female **G** tibia and tarsus IV of female.

**Female.** (Range for 3 paratypes) (Figs 3, 4, 5F, G). Length of idiosoma from anterior end to bases of setae *h3* 425–440, greatest width 190–200, length of hysterosoma 270–275. Subcapitulum shaped as in male. Prodorsal shield: shaped almost as in male, except posterior margin blunt-angular and extending beyond level of setae *si*, 85–92 long, 58–62 wide (Fig. 3). Setae *se* separated by 85–88. Scapular shields more narrow than in male. Humeral shields not developed. Setae c2 short filiform, situated on soft tegument. Setae *cp* situated ventrally on soft tegument, 135–150 long. Hysteronotal shield: large longitudinal plate occupying median part of hysterosoma; anterior margins almost straight, extending to or beyond level of setae *cp*; lateral margins slightly concave; posterior margin deeply concave, posterior angles encompassing bases of setae e2; greatest length 185–190, greatest width 74–83. Setae *d1*, *e1* and *e2* situated on hysteronotal shield, setae *d2* situated on striated tegument. Distance between dorsal setae: *c2:d2* 92–95, *d2:e2* 112–118, *e2:h3* 50–55, *d2:d2* 91–93, *e2:e2* 71–75, *h2:h2* 59–65, *h3:h3* 49–50.

Epimerites I fused into a Y, stem about half as long as epimerites. Epigynum shaped as thick bow-shaped transverse bulk with a pair of acute posterior branches, 29–34 long, 56–63 wide. Apodemes of oviporus long, posterior ends extending to midlevel of trochanters III (Fig. 4). Seta *4a* situated on epigynum. Setae *4b*, *g*, *3a*, and *4a* short filiform, not exceeding length of femorogenua III, IV. Setae *h3* slightly shorter than setae *h2*.

Legs I, II as in the male. Leg IV with distal half of tarsus extending beyond posterior end of opisthosoma. Tarsi III, IV without apical spines. Length of tarsi III and IV 73–75 and 81–84, respectively. Lengths of solienidia: *σ*I 67–71, *σ*II 24–33, *σ*III 49–52, *φ*III 57–67, *φ*IV 91–100 (Fig. 5F, G).

##### Differential diagnosis.

The genus *Mycterialges* has included only the type species, *Mycterialgesmesomorphus*, and one additional undescribed species (Gaud and Atyeo 1981a; Gaud 1982). The new species *Mycterialgesboycianae* sp. nov. differs from *M.mesomorphus* by a number of characters: in males of *M.boycianae*, the prodorsal shield consists of a single triangular plate, the anterior part of the hysteronotal shield is widened and has a pair of narrow extensions, the paragenital apodemes are fused into a large teardrop sclerite encompassing genital apparatus, the legs IV are much shorter than legs III, and tibia + tarsus IV are shorter than femoragenu IV, tarsus IV is conical and ambulacral disc of tarsus IV is absent; in females, the posterior margin of prodorsal shield is blunt-angular and extends slightly beyond the level of setae *si*, the hysteronotal shield is as wide as the prodorsal shield and its posterior margin is deeply concave, setae *e2* are situated on the posterior corners of the hysteroronotal shield, and setae *g* and *3a* are situated on the same transverse level. In males of *M.mesomorphus*, the prodorsal shield consists of two plates (triangular anterior parts and trapezoidal posterior part), the anterior part of the hysteronotal shield is narrowed and without extensions, the paragenital apodemes are fused into ovate sclerites around genital apparatus and with a pair of posterior projections, the legs IV are almost as long as legs III, and tibia + tarsus IV are much longer than femoragenu IV, tarsus IV has a claw-like apical process, and ambulacral disc of tarsus IV is narrowly lanceolate; in females, the posterior margin of prodorsal shield is straight and does not extend to the level of setae *si*, the hysteronotal shield is narrow, approximately half as wide as the prodorsal shield and its posterior margin is straight, setae *e2* are situated on the soft tegument at the level of the posterior margin of the hysteronotal shield, and setae *g* are anterior to level of setae *3a*. Actually, *M.mycterialges* sp. nov. is much more similar to the unnamed *Mycterialges* species, known only from male and illustrated but not described (Gaud 1982: fig. 6a, b), in sharing the following features: the prodorsal shield is triangular, the anterior end of the hysteronotal shield has a pair of narrow extensions, legs IV are much shorter that legs III, and tibia+tarsus IV are shorter than femoragenu IV, tarsus IV is conical, and ambulacral disc of pretarsus IV is absent. Males of *M.boycianae* differs from those of the unnamed *Mycterialges* species in the following features: setae *d2* are represented by macrosetae extending beyond the posterior margin of opisthosoma, setae *cp* are situated on the humeral shields, and the paragenital apodemes fused into the teardrop-shaped sclerite free from epimerites IV. In the male of the unnamed *Mycterialges* species, setae *d2* extend to midlevel between setae *e2* and *h2*, setae *cp* are situated on striated tegument, and the paragenital apodemes fused into the teardrop-shaped sclerite are fused with the inner tips of epimerites IV.

**Figure 6.**
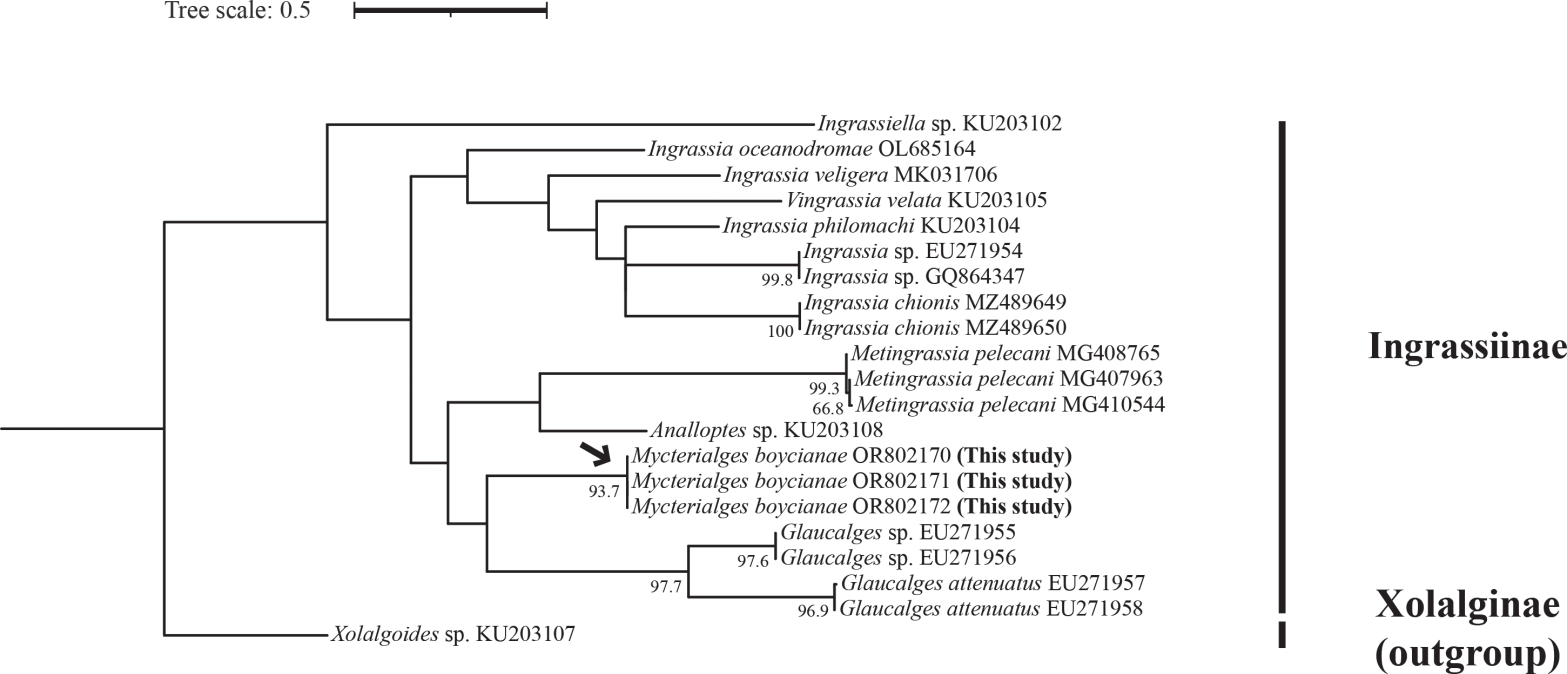
Maximum-likelihood phylogenetic tree of COI barcode fragment for members of the subfamily Ingrassiinae. Bootstrap percentages of more than 50% are shown. Scale bars indicate the number of substitutions per nucleotide site. The subfamily Xolalginae is the outgroup taxon.

##### Etymology.

The specific name is taken from the species epithet of the type host and is a noun in the genitive case.

### ﻿Phylogenetic relationships based on mtDNACOI

The ML phylogenetic tree of the COI barcode fragment showed *Mycterialgesboycianae* sp. nov. to be grouped within the subfamily Ingrassiinae clade with three individuals of *M.boycianae*, exhibiting 93.7% bootstrap support for this grouping. Among the COI sequences collected from NCBI, the genus *Glaucalges* formed the clade closest to *M.boycianae* (Fig. 6). The genetic distance between *M.boycianae* and *Glaucalges* species was estimated to be ~16.1–16.2%.

## ﻿Discussion

This study describes a new feather mite species, *Mycterialgesboycianae* sp. nov. found on the wings and body of captive *Ciconiaboyciana* at the Yesan Oriental Stork Park in Korea. The genus *Mycterialges* has the following features: in both sexes, tarsi I, II only have two and one ventral setae, respectively, and the ambulacral disc is inverted triangle-shaped with a concave middle; in males, the opisthosomal lobes are fused and bluntly rounded, leg IV is hypertrophied; in females, the epigynum is short and straight (Gaud and Atyeo 1981a, 1996). *Mycterialgesboycianae* has all these main characteristics (Figs 1–5), but exhibits significant variation from the type species in the legs IV. In the case of the type species *M.mesomorphus*, the male legs IV are hypertrophied, but not to the terminal width, and there is an ambulacrum (Gaud and Atyeo 1981a). In contrast, legs IV of *M.boycianae* and *M.* sp. (Gaud and Atyeo 1981a, 1996), are hypertrophied to the extent of the terminal width, have a femorogenu that combines the femur and genu, and have a claw instead of an ambulacrum (Figs 1–2, 5) (Gaud and Atyeo 1996). Despite these significant differences, no other species have been identified in *Mycterialges* apart from the type species so far. Therefore, we have included the newly discovered feather mite in *Mycterialges* based on the form of *M.* sp., despite the lack of precise identification by Gaud and Atyeo (1981a, 1996). We observed that the mites discovered in the genus *Mycterialges* exhibit significant differences in legs IV, thus suggesting that there is a need to redefine this genus.

We showed the phylogenetic relationship of Ingrassiinae using the COI barcode fragment (Fig. 6). However, despite gathering all available Ingrassiinae data from the NCBI, our tree could only provide minimal information. This is due to the fact that, while the Ingrassiinae consists of 16 genera and 106 species (Gaud and Atyeo 1981a; Mironov and Galloway 2002; Mironov et al. 2005; Dabert et al. 2008; Constantinescu et al. 2013; Stefan et al. 2013; Hernandes 2014; Li and Zhang 2016; Mironov et al. 2017; Hernandes and Pedroso 2017; Han et al. 2021; Hernandes and Brito 2022), our tree data includes only seven genera and 12 species, some of which have not been accurately described. Therefore, it is necessary to collect more data on mites to obtain more accurate results (Maddison and Knowles 2006; Knowles and Klimov 2011).

Finally, we discuss the situations faced by *M.boycianae*. This mite’s host, *C.boyciana*, is not only an endangered species in Korea, but is also listed as an ‘Endangered’ (EN) species on the International Union for Conservation Nature (IUCN) Red List (BirdLife International 2018). Moreover, the nesting sites of the Oriental Stork are extremely limited and confined to parts of Russia and China (Zhou et al. 2013; BirdLife International 2018). Given its high host specificity, this situation could be extinction-threatening for *M.boycianae*, a permanent ectosymbiont (OConnor 1982; Doña et al. 2019). Therefore, we believe that it is necessary to investigate the distribution, biology, ecology, and conservation status of feather mites, including *M.boycianae*, to confirm whether ectosymbionts are at risk of extinction.

## Supplementary Material

XML Treatment for Mycterialges

XML Treatment for Mycterialges boycianae

## ﻿Additional information

### Author ORCIDs

Jeong Hun Shim https://orcid.org/0009-0000-1858-7725

Yeong-Deok Han https://orcid.org/0000-0002-6625-8966

Sukyung Kim https://orcid.org/0000-0003-3545-0282

Dongsoo Ha https://orcid.org/0000-0002-1943-9190

Yongun Shin https://orcid.org/0000-0001-7491-994X

Soo Hyung Eo https://orcid.org/0000-0001-6719-1612

### Data availability

All of the data that support the findings of this study are available in the main text.
